# Experimental Infection of Cynomolgus Macaques (*Macaca fascicularis)* with Aerosolized Monkeypox Virus

**DOI:** 10.1371/journal.pone.0012880

**Published:** 2010-09-20

**Authors:** Aysegul Nalca, Virginia A. Livingston, Nicole L. Garza, Elizabeth E. Zumbrun, Ondraya M. Frick, Jennifer L. Chapman, Justin M. Hartings

**Affiliations:** 1 Center for Aerobiological Sciences, United States Army Medical Research Institute of Infectious Diseases (USAMRIID), Fort Detrick, Maryland, United States of America; 2 Pathology Division, United States Army Medical Research Institute of Infectious Diseases (USAMRIID), Fort Detrick, Maryland, United States of America; Naval Medical Research Center Detachment, Peru

## Abstract

Monkeypox virus (MPXV) infection in humans results in clinical symptoms very similar to ordinary smallpox. Aerosol is a route of secondary transmission for monkeypox, and a primary route of smallpox transmission in humans. Therefore, an animal model for aerosol exposure to MPXV is needed to test medical countermeasures. To characterize the pathogenesis in cynomolgus macaques (*Macaca fascicularis)*, groups of macaques were exposed to four different doses of aerosolized MPXV. Blood was collected the day before, and every other day after exposure and assessed for complete blood count (CBC), clinical chemistry analysis, and quantitative PCR. Macaques showed mild anorexia, depression, and fever on day 6 post-exposure. Lymphadenopathy, which differentiates monkeypox from smallpox, was observed in exposed macaques around day 6 post-exposure. CBC and clinical chemistries showed abnormalities similar to human monkeypox cases. Whole blood and throat swab viral loads peaked around day 10, and in survivors, gradually decreased until day 28 post-exposure. Survival was not dose dependent. As such, doses of 4×10^4^ PFU, 1×10^5^ PFU, or 1×10^6^ PFU resulted in lethality for 70% of the animals, whereas a dose of 4×10^5^ PFU resulted in 85% lethality. Overall, cynomolgus macaques exposed to aerosolized MPXV develop a clinical disease that resembles that of human monkeypox. These findings provide a strong foundation for the use of aerosolized MPXV exposure of cynomolgus macaques as an animal model to test medical countermeasures against orthopoxviruses.

## Introduction

After a centuries-long battle, humans defeated smallpox and it was declared eradicated in 1980. Smallpox vaccination was then discontinued, leaving most of today's population vulnerable to *Variola* virus (VARV) (smallpox agent). MPXV is a close relative of VARV, sharing 96.3% identity within the central region of the genome encoding essential genes, and 84.5% identity overall [1]. MPXV causes a disease in humans that is clinically indistinguishable from ordinary smallpox, with the exception of lymphadenopathy [1], [2], [3]. Vaccinia-based vaccines, used for worldwide eradication of VARV, are protective against MPXV challenge in animal models and are also presumed to protect humans from monkeypox [4], [5], [6], [7], [8], [9], [10], [11], [12]. The similarity of monkeypox to smallpox, and the growing lack of immunity in the population have caused concerns that these viruses might be used as biological weapons. This has prompted scientists to develop new medical countermeasures against poxviruses. Because evaluation of medical countermeasures against poxvirus infection in humans is not ethical or feasible, showing efficacy in an animal model that emulates human disease is required by the “Animal Rule” of the US Food and Drug and Administration (FDA) [13]. Non-human primates (NHPs) are closely related to humans and are often the most accurate model system for the study of human disease processes. Therefore it is important to develop a model of MPXV infection in NHPs, using the most relevant route, in order to fully evaluate pathogenesis as well as the capabilities of vaccines and therapeutics.

The use of VARV in research is highly restricted; therefore, viruses from other members of the orthopoxvirus family are used to develop animal models to test medical countermeasures against poxviruses. There are few orthopoxvirus animal disease models that simulate the pathophysiology and unique clinical progression of smallpox and monkeypox in humans. The current models utilize a wide range of orthopoxviruses, animal species, and challenge routes. The current animal models include: vaccinia virus in mice by intranasal (i.n.), intraperitoneal (i.p.) or intravenous (i.v.) routes, cowpox virus in mice and marmosets by the i.n. route, ectromelia in mice by aerosol and i.n. routes, vaccinia virus or rabbitpox virus in rabbits by intradermal (i.d.) or aerosol routes, monkeypox virus in dormice, prairie dogs or ground squirrels by i.n. or i.p. routes, monkeypox virus in monkeys by intratracheal (i.t.) and i.v. routes, and VARV in monkeys by the i.v. route +/− aerosol route [12], [14], [15], [16], [17], [18], [19], [20], [21].

It is well established that VARV is transmitted by the aerosol route [22]. Furthermore, an intentional release of VARV or MPXV would likely be in aerosol form. Therefore, the aerosol route of transmission should be one of the features of an animal model which will be used as a model for human smallpox and monkeypox infection. No single model recapitulates all the aspects of smallpox or monkeypox in humans, yet the most relevant models, MPXV or VARV infection of NHPs by the aerosol route, have not been fully characterized [23], [24], [25]. Studies of aerosol MPXV infection models require biosafety level 3 (BSL-3) laboratories and class III biosafety cabinets containing specialized aerosol equipment. The single published study of aerosolized MPXV infection of cynomolgus macaques gave a detailed account of the pathology induced by the virus in various tissues, but did not address a number of facets of the clinical disease progression [23], [25]. We present that cynomolgus macaques exposed to aerosolized MPXV show many characteristics of monkeypox and smallpox in humans and is thus an appropriate model for orthopoxvirus pathogenesis, vaccine and therapeutic studies.

## Materials and Methods

### Animals and Ethical Statement

Healthy, adult cynomolgus macaques (*Macaca fascicularis*) of both sexes were obtained from the United States Army Medical Research Institute of Infectious Diseases (USAMRIID) NHP colony. All MPXV exposed animals were handled in a BSL-3 containment laboratory at USAMRIID. Research was conducted in compliance with the Animal Welfare Act and other federal statutes and regulations relating to animals and experiments involving animals, and adhered principles stated in the Guide for the Care and Use of Laboratory Animals, National Research Council, 1996. The facility where this research was conducted (USAMRIID) is fully accredited by the Association for the Assessment and Accreditation of Laboratory Animal Care International. Research was conducted under a protocol approved by the Institutional Animal Care and Use Committee (IACUC) at USAMRIID. All animals were examined and evaluated twice per day by study personnel. Early endpoint criteria, as specified by the score parameters within the “Post-exposure observations” section of these methods, were used to determine when animals should be humanely euthanized.

### Virus

MPXV (Zaire V79-I-005 strain) was provided by the Biodefense and Emerging Infections Research Resources Repository (BEI Resources)/ATCC. Virus was diluted in Eagle's Minimum Essential Medium (EMEM) with 2% fetal bovine serum (FBS) to achieve desired doses (Table 1).

**Table 1:** Summary of inhaled doses, fever, and disease outcome in cynomolgus macaques exposed to aerosolized MPXV.

Groups (PFU)	Inhaled dose (PFU) (average)	Fever onset (study day)^a^	Fever duration (hours)^b^	Fever hours c	ΔT _max_, °C d	Average elevation in temperature (°C) e	MTD (days) f	Survivors/total
4×10^4^	4.3×10^4^	4.7	105.3	215.3	2.5	1.9	10.0	1/3
1×10^5^	1.4×10^5^	3.8	121.5	244.7	3.3	1.9	9.0	2/6
4×10^5^	4.4×10^5^	2.8	120.8	266.7	3.4	2.1	9.6	1/6
1×10^6^	1.1×10^6^	4.3	123.6	278.1	3.5	2.3	8.5	1/3

a Defined as the first day with >8 h of significant temperature elevation (as determined by ARIMA modeling).

b Calculated as the number of days (converted to hours) with 12 or more h of significant temperature elevation.

c Calculated as the sum of the significant temperature elevations.

d The maximum change in temperature.

e Calculated by dividing fever hours by fever duration in hours.

f Mean time-to-death.

### Plaque Assay

MPXV was titrated in complete Eagles Minimum Essential Media containing non-essential amino acids (EMEM/NEAA media) supplemented with 2% FBS, penicillin (8 IU/mL), streptomycin (80 µg/mL), gentamicin (0.02 mg/mL) and fungizone (0.1 U/mL) warmed to 37°C. Plaque assays were carried out on Vero E6 cells at approximately 95% confluency. One hundred µL of each dilution was added to each well of a six-well plate. Plates were incubated for 1 hour in at 37°C, rocking plates every 10–15 minutes. After one hour, two mL of complete media were added to each well and incubated for 4 days at 37°C. On day 4, media was removed from the plates and 500 µL 10% crystal violet was added to each well for approximately 10–20 minutes. Once cells were stained, plates were rinsed by submersion in cold water and placed upside down to dry overnight. Plaques were counted the following day.

### Aerosol exposures

Each macaque was anesthetized by intramuscular (i.m.) injection of tiletamine/zolazepam (6 mg/kg), and whole body plethysmography (Buxco Research Systems, Wilmington, NC) was performed to determine the respiratory minute volume as previously described [26]. Subsequently, each macaque was exposed to MPXV in a head-only chamber contained in a class III biological safety cabinet located inside a BSL-3 suite. The Automated Bioaerosol Exposure System (ABES) served as the control platform for the aerosol exposures [27]. Aerosols were generated with a three-jet collison nebulizer (BGI, Inc., Waltham, MA), and integrated air samples were collected throughout the exposure with an all glass impinger (AGI).

To improve the precision of presented aerosol doses, the ABES was programmed to dynamically calculate the exposure time based on the minute volume measurement for each macaque, the flow to volume ratio of the exposure chamber, the starting MPXV concentration in the collison nebulizer, and the historical spray factor for MPXV virus. For each individual exposure, these parameters were entered into the ABES, and the ABES determined the time required to reach the required dose. ABES calculations were based on a dynamic model that estimates chamber aerosol concentration based on the flow to volume ratio in the chamber.

After exposure, AGI samples were analyzed by performing a plaque assay. An inhaled MPXV dose was calculated for each macaque based on the plaque assay and the minute volume measurement.

### Telemetry

A radiotelemetry device (Data Sciences International (DSI), St. Paul, MN), used to monitor temperature and activity, was surgically implanted into macaques at least 14 days before aerosol exposure. Body temperatures were recorded every 15 min by the DataQuest A.R.T.4.1 system (DSI). Pre-exposure temperature data were used to create a baseline to fit an autoregressive integrated moving average (ARIMA) model. Temperature elevations exceeding three standard deviations over the baseline were used to compute fever duration, hours, and average elevation.

### Post-exposure observations

Macaques were observed at least twice a day after aerosol exposure. Macaques were scored for clinical signs of disease prior to, and while under anesthesia. The scoring parameters were: responsiveness and appearance (0: active; 2: depression, mild unresponsiveness; 3: head down, hunched; 4: moderate unresponsiveness; 5: severe unresponsiveness), dyspnea (0: normal breathing; 2: mildly labored; 3: labored; 5: agonal breathing), dehydration (0: not present; 1: mild; 2: moderate; 3: severe), anorexia (0: eating; 1: no biscuits for 1 day but eats enrichment; 2: no biscuits for 2 days or not eating enrichment), rash (0: none; 1: slight; 2: moderate; 3: severe), cough (0: none; 1:≤2 coughs/5 min; 2: 3–10 coughs/5 min; 3: ≥10 coughs/5 min), nasal discharge (0: none; 1: mild; 2: moderate; 3: severe), urine (0: normal; 3: none), stool (0: normal; 1: loose stool, 2: liquid stool or none), and fever (0: no change, 1: baseline +1°C; 2: baseline +2°C or higher; 3: baseline – 2°C). Macaques were also evaluated for changes in weight (0: no change; 1: baseline-10–15%; 2: baseline – 15%; 3: baseline – 20%) and the presence of lymphadenopathy (0: <3 mm; 1: 3–9 mm; 2: >10–19 mm; 3: >20 mm). The early endpoint criteria for humane euthanasia, indicative of very poor health status, were cumulative clinical scores of 15–20 (maximum score), and/or a sudden drop of >3°C from baseline body temperature.

### Clinical laboratory evaluations

Beginning one day before, and every other day on days 2–28 after exposure, blood samples were collected from the femoral vein of macaques anesthetized with tiletamine/zolazepam (3 mg/kg; IM). Samples collected one day prior to exposure served as a normal reference baseline for each animal. CBCs and blood chemistries were analyzed with Beckman Coulter hematology and VITROS 250 chemistry analyzers.

### DNA extraction and real-time polymerase chain reaction (RT-PCR)

Tissue samples were collected from all major organ systems and frozen. One gram of each tissue was pulverized with 1 mL EMEM plus 2.5 mL penicillin (20,000 IU/mL)/streptomycin (200,000 µg/mL) solution using a handheld Omni International (Kennesaw, GA) tissue homogenizer with a single use disposable plastic tissue grinding tip. Blood and throat swabs were collected from macaques every other day after exposure to aerosolized MPXV. Viral DNA was isolated from 200 µl of tissue suspension, 100 µl of blood, or 100 µl of throat swab media with a BioRobot M48 (Qiagen, Valencia, CA) according to the manufacturer's instructions. Real-time PCR was performed with the LightCycler (Roche, Indianapolis, IN) using a pan-orthopoxvirus hemagglutinin assay (HA), as previously described [28].

### Necropsy

A necropsy was performed under BSL-3 conditions on animals that were humanely euthanized when moribund or, if they survived, at the end of the study. Tissue samples from all major organ systems (respiratory, gastrointestinal, genitourinary, lymphoid, neurologic, endocrine, skin and mucous membranes) were collected from each animal for histopathological and immunohistochemical examination and were immersion-fixed in 10% neutral-buffered formalin.

### Histology and immunohistochemistry

Formalin-fixed tissues for histologic examination were trimmed, processed, and embedded in paraffin according to established protocols [29]. Histology sections were cut at 5 µm, mounted on glass slides, and stained with hematoxylin and eosin (H&E). Immunohistochemical staining was performed on replicate tissues sections using an EnVision + kit (DAKO, Carpinteria, CA). Normal splenic tissue served as the negative control; the positive control was spleen from a known MPXV infected NHP; and normal rabbit serum was used as the negative serum control.

Briefly, sections were deparaffinized in xyless, rehydrated in graded ethanol, and endogenous peroxidase activity was quenched in a 0.3% hydrogen peroxide/methanol solution for 30 min at room temperature. Slides were washed in phosphate buffered saline (PBS) then sections were incubated in the primary antibody, a rabbit polyclonal antibody against vaccinia virus, diluted 1∶3500 for 60 minutes at room temperature. Sections were washed in PBS and incubated for 30 min with EnVision + rabbit secondary reagent (horseradish peroxidase-labeled polymer) at room temperature. Peroxidase activity was developed with 3, 3′-diaminobenzidine (DAB), counterstained with hematoxylin, dehydrated, cleared with xyless, then coverslipped.

### Digital Microscopy Image Analysis

Digital microscopy was performed using an Automated Cellular Imaging System (ACIS® II, Dako, Carpenteria, CA) which uses proprietary software to allow for color detection and analysis of morphometric features. This system consists of an automated robotic bright-field microscope module, a computer, and a Microsoft Windows NT based software interface. The robotic microscope module scanned the immunohistochemically stained slides and the digitized images were displayed on the computer monitor. The pathologist reviewed the images and an ACIS-assisted score was generated by the system software as a means of quantitating the vaccinia virus staining in the tissue. Technical details about the ACIS digital microscopy system are presented elsewhere [30].

### Statistical analysis

Repeated measures analysis of variance (RM-ANOVA) was used to compare temperature, weight, white blood cells (WBC), blood chemistries, and viral genomes among groups until day 10, since after that time, the 1×10^5^ PFU group had two survivors and the other groups had one survivor each. Group comparisons for time-to-death were calculated by t-tests with step-down Bonferrini correction. RM-ANOVA of log_10_ transformed data was used for comparison of viral genome load in blood and throat swabs between groups over time. T-tests of log_10_ transformed data were performed for viral genome load from tissues between groups. Analyses were two-tailed and conducted using SAS v9.1.3. Results were significant at *P*<0.05.

## Results

### Clinical signs and survival

In this study, four groups of cynomolgus macaques were exposed to increasing doses of aerosolized MPXV, with a particle size of 1–3 µm (Table 1). As little as 200 pfu MPXV delivered by the aerosol route was sufficient to cause non-lethal disease, including lesions (data not shown). The doses were 4×10^4^ PFU (n = 3), 1×10^5^ PFU (n = 6), 4×10^5^ PFU (n = 6), and 1×10^6^ PFU (n = 3), chosen based on optimization of the dose range required for consistent infection (data not shown). The calculated inhaled doses were very close to target doses for each group (Table 1).

**Figure 1:**
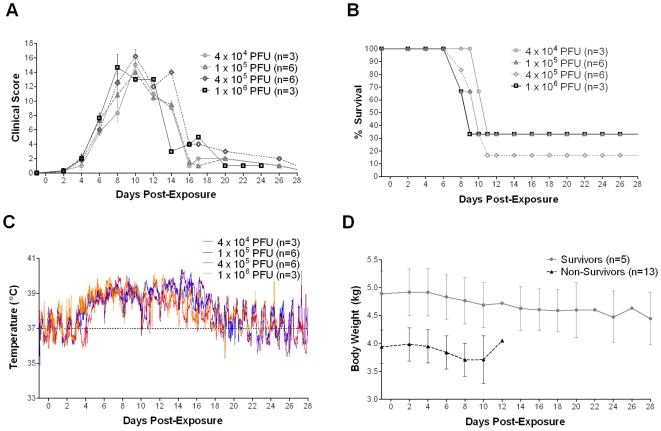
Clinical disease scores, survival, temperature and weight. **A**) Changes in average clinical scores and **B**) percent survival of macaques exposed to different doses of aerosolized MPXV. **C**) Average body temperature of macaques exposed to different doses of aerosolized MPXV. The temperature data was collected every 15 min from implanted telemetry devices. The dashed line indicates the average baseline body temperature; n: number of animals. **D**) Changes in average body weight of survivors and non-survivors exposed to aerosolized MPXV. n: number of animals.

Animals started to show clinical signs of disease, including decreased appetite and activity, by day 3. Lymphadenopathy of inguinal and axillary nodes was observed starting 6–7 days post-exposure. By 6–8 days post-exposure, macules began to form in all animals and macaques were also inactive, somnolent, and exhibited depressed posture. The clinical score of all macaques peaked on day 10 post-exposure and there were no significant differences among groups (Figure 1A). Lesions progressed to papules by day 10 and evolved to vesicular and pustular stages by 12–14 days post-exposure. Surviving macaques were active, eating well, had scabbed lesions and had greatly reduced clinical scores by day 20. Of note, the number of lesions was not dose dependent and varied widely with 60–730 lesions among survivors, and 10–180 lesions among 11 of 13 non-survivors. Two non-survivors had too many lesions to count (>2000).

Survival was not dose dependent, with doses of 4×10^4^ PFU, 1×10^5^ PFU, and 1×10^6^ PFU resulting in 33% survival and 4×10^5^ PFU resulting in approximately 17% survival (Fig. 1B). Although death was delayed in the lowest dose group, there were no significant differences in the mean time-to-death (MTD) (Table 1). Most of the macaques met criteria for euthanasia on days 8–11. Surviving macaques had less severe disease, except for one macaque in the highest dose group, which had severe disease but never met the score criteria for euthanasia.

### Body temperature and weight changes

Fever was delayed until day 5 in the lowest dose group compared to day 4 for the other groups (Table 1 and Figure 1C). However, differences were not significant between the groups regarding onset, duration, or magnitude of fever. By day 5 post-exposure, all groups had an average elevation in body temperature of 2°C. The lowest dose group had the shortest fever duration, whereas the highest dose group had the longest. Similarly, the lowest dose group had the fewest fever hours, calculated as the sum of significant temperature elevations, and the highest dose group had the most.

Even though there were no significant weight changes over time among groups after MPXV exposure, weight was significantly different between survivors and non-survivors regardless of dose (*P*<0.0001) (Figure 1D). Survivors were approximately 20% heavier than non-survivors.

### Clinical laboratory evaluation

**Figure 2:**
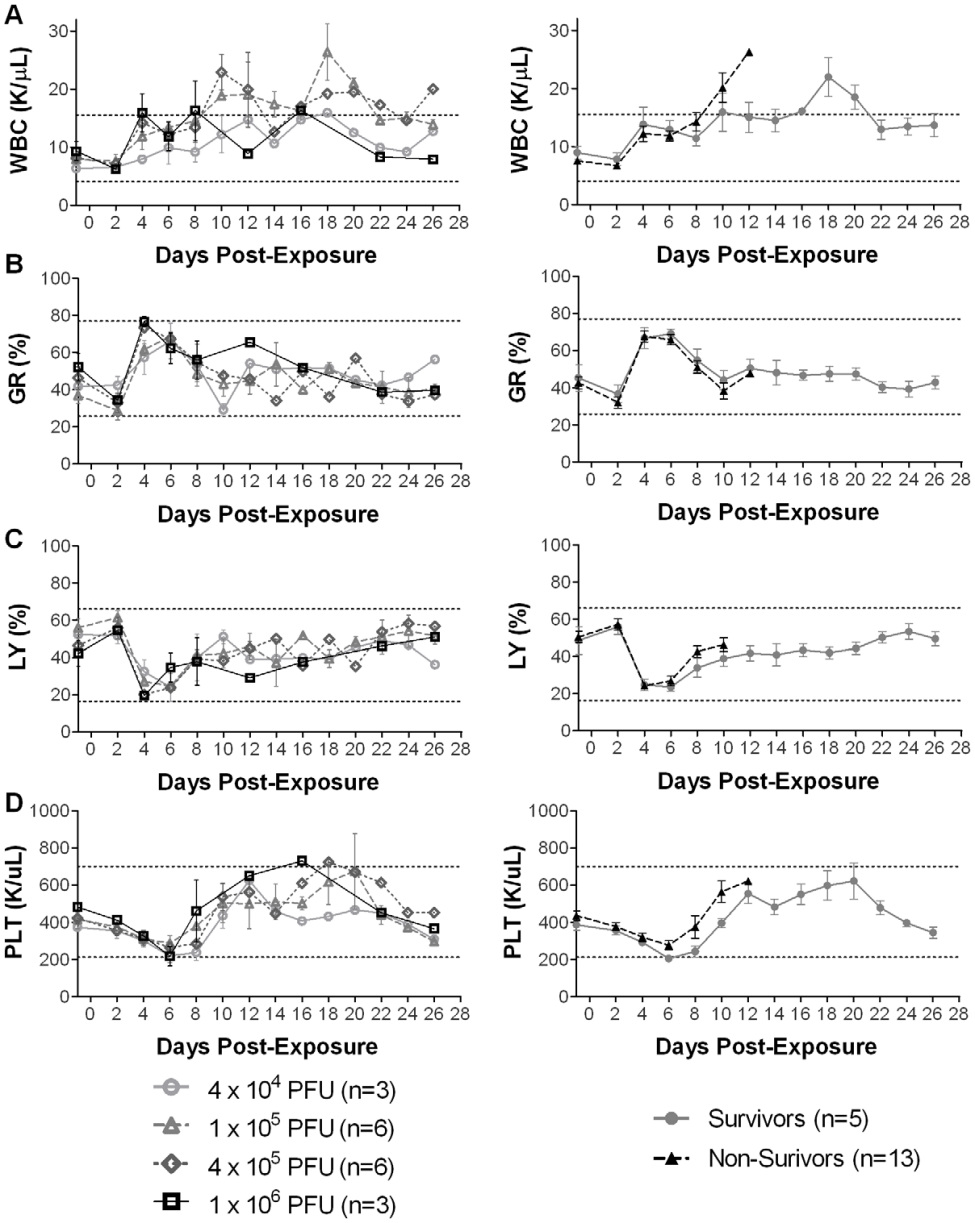
Average number of leukocytes and platelets in macaques after exposed to aerosolized MPXV. The dotted lines indicate the normal reference range; n: number of animals. Graphs are shown for **A**) total white blood cells (WBC), **B**) percentage of granulocytes (GR), **C**) percentage of lymphocytes (LY), **D**) platelets (PLT) for all MPXV dosage groups, and survivors versus non-survivors (right).

In contrast to a previous report on aerosol infection of cynomolgus macaques with MPXV which reported no significant changes in CBCs or blood chemistries after infection, a number of changes were observed in this study [25]. WBCs decreased slightly on day 2 and increased after day 4 in all groups except for the lowest dose group (*P = *0.0011) (Fig. 2A). All three dosage groups had data points that were above the normal reference range on certain days. Granulocytes decreased on day 2, followed by a sharp increase on day 4 post-exposure (Fig. 2B). In contrast, peripheral lymphocytes increased on day 2 followed by a sharp decrease on day 4 post-exposure (Fig. 2C). There were no significant differences on days −1–10 between survivors and non-survivors regarding changes in WBC, granulocyte, or lymphocyte values.

Platelets decreased steadily from day 2 to day 6. Platelets were lowest on day 6 post-exposure, which was significantly lower than day −1 (*P*<0.0001), and increased after day 6 but stayed within the normal range (Fig. 2D). Interestingly, non-survivors had significantly higher levels of platelets than survivors on days −1 to 10 (*P = *0.0011).

**Figure 3:**
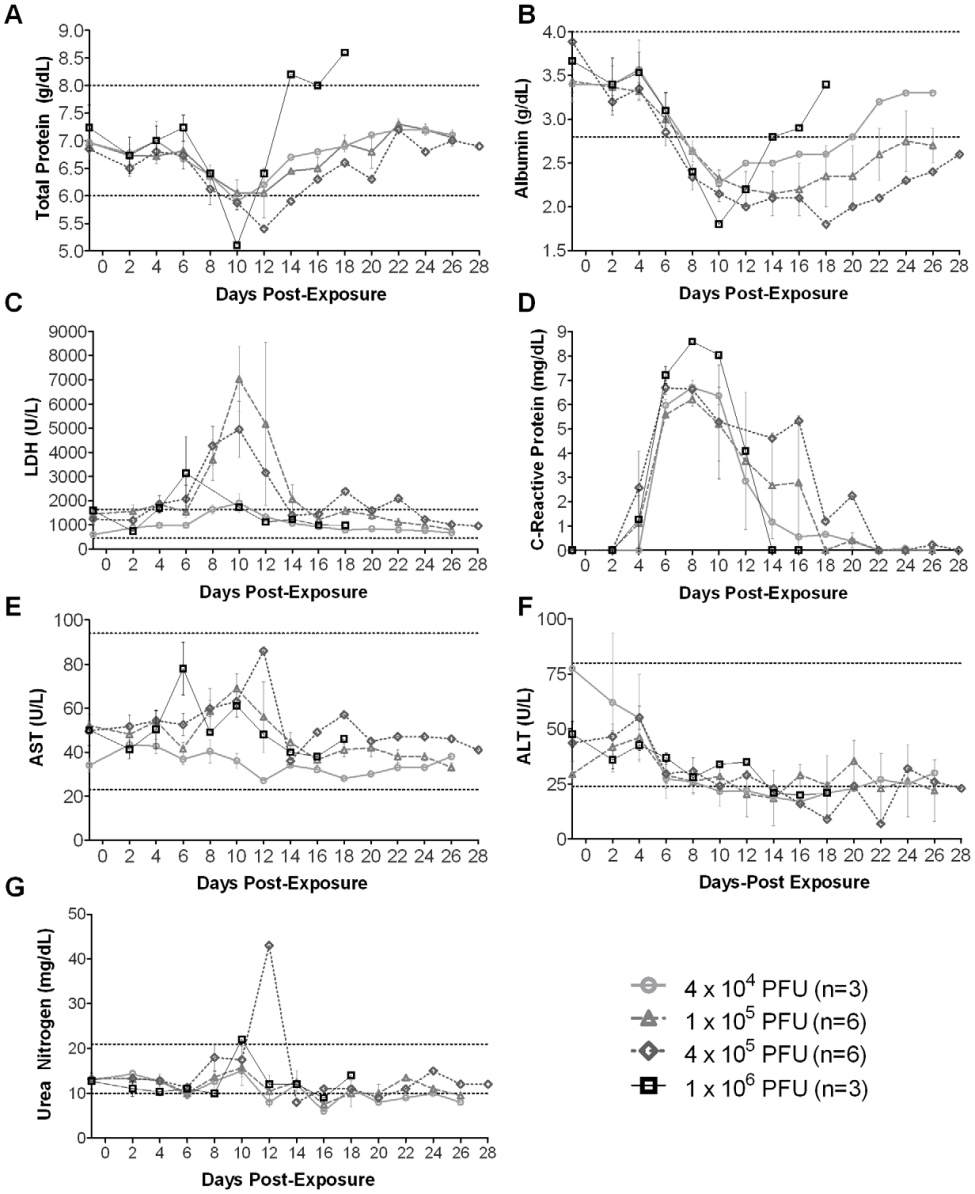
Serum chemistries in macaques exposed to aerosolized MPXV. The dotted lines indicate the normal reference range; n: number of animals. Graphs show average **A**) total protein, **B**) albumin, **C**) lactate dehydrogenase (LDH), **D**) C-reactive protein, **E**) aspartate transaminase (AST), **F**) and alanine transaminase (ALT), **G**) urea nitrogen.

Serum chemistries were evaluated for all macaques one day before exposure and every other day on days 2–28. Considerable changes were observed in total protein, albumin, lactate dehydrogenase (LDH), and C-reactive protein values in all macaques over time, but the changes were similar for all groups on days −1 to 10 for total protein, albumin, and C-reactive protein (Fig. 3A-D). There were also no significant differences in serum chemistries on days −1–10 between survivors and non-survivors (data not shown). In contrast, there were significant differences in LDH levels between groups after MPXV infection (*P = *0.0002). Alanine and aspartate amino transaminases (ALT and AST) stayed within normal ranges and groups did not differ statistically (Fig. 3E-F). Levels of blood urea nitrogen (BUN) did not differ significantly among groups (Fig. 3G). However, compared to day -1, BUN was significantly lower for all animals on day 6 (*P = *0.0003), and higher for animals surviving until day 10 (*P = *0.0091).

### Viral load

**Figure 4:**
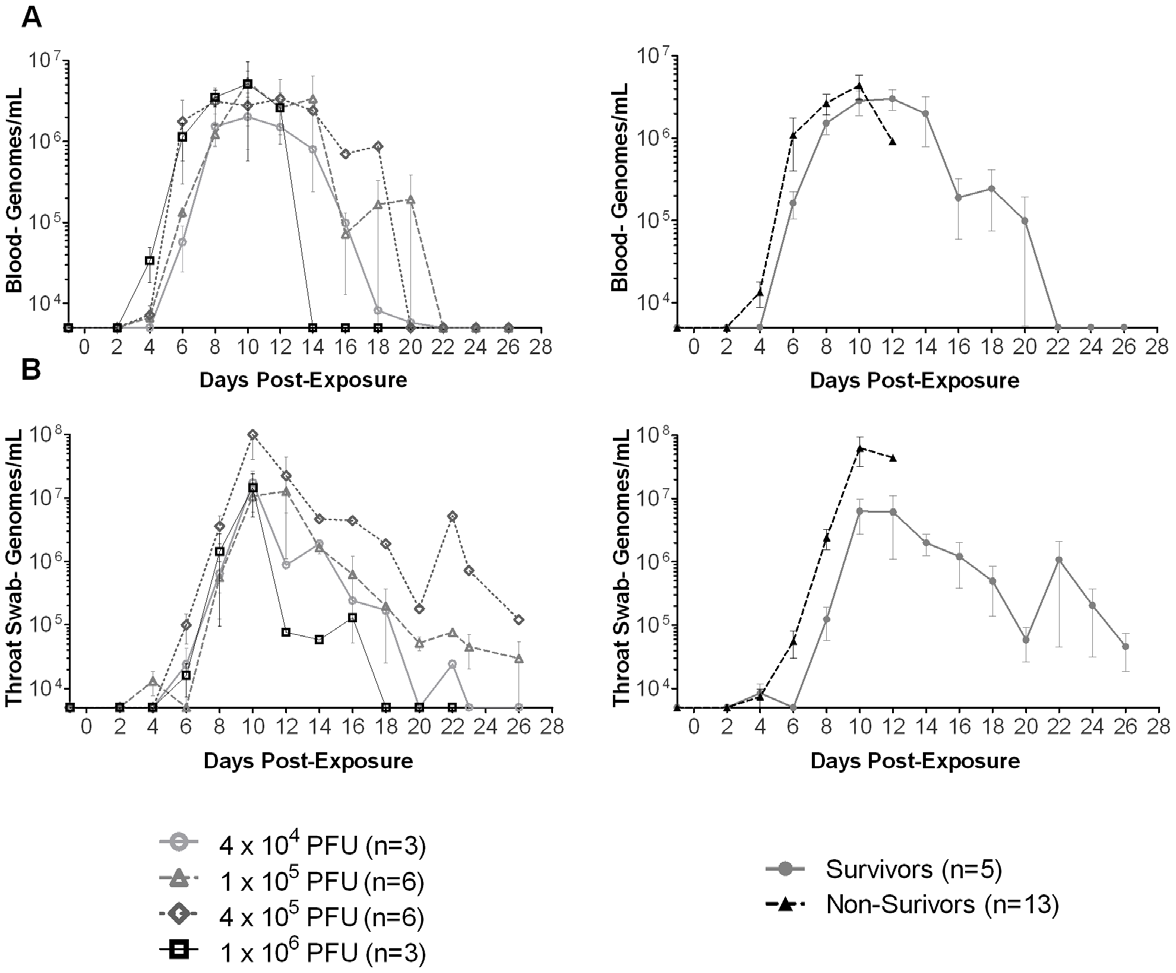
Average number of MPXV viral genomes in whole blood and throat swabs. The average number of MPXV viral genomes in **A**) whole blood, and **B**) throat swabs from different MPXV dosage groups, and survivors versus non-survivors (right). The X-axis is at the limit of detection  = 5000 genomes/ml; n: number of animals.

Viral loads in whole blood and throat swab samples from exposed macaques were assessed using real-time PCR (Figure 4A-B). Viral genomes in both whole blood and throat swabs were detected as early as day 4 post-exposure. The viral load peaked on day 10 for both blood and throat swab samples and gradually decreased thereafter. There were no group-wise differences between blood or throat swab viral load on days 1–10, but the viral levels in whole blood and throat swabs were higher in non-survivors than survivors (blood *P = *0.0445, throat swabs *P*<0.0001).

**Figure 5:**
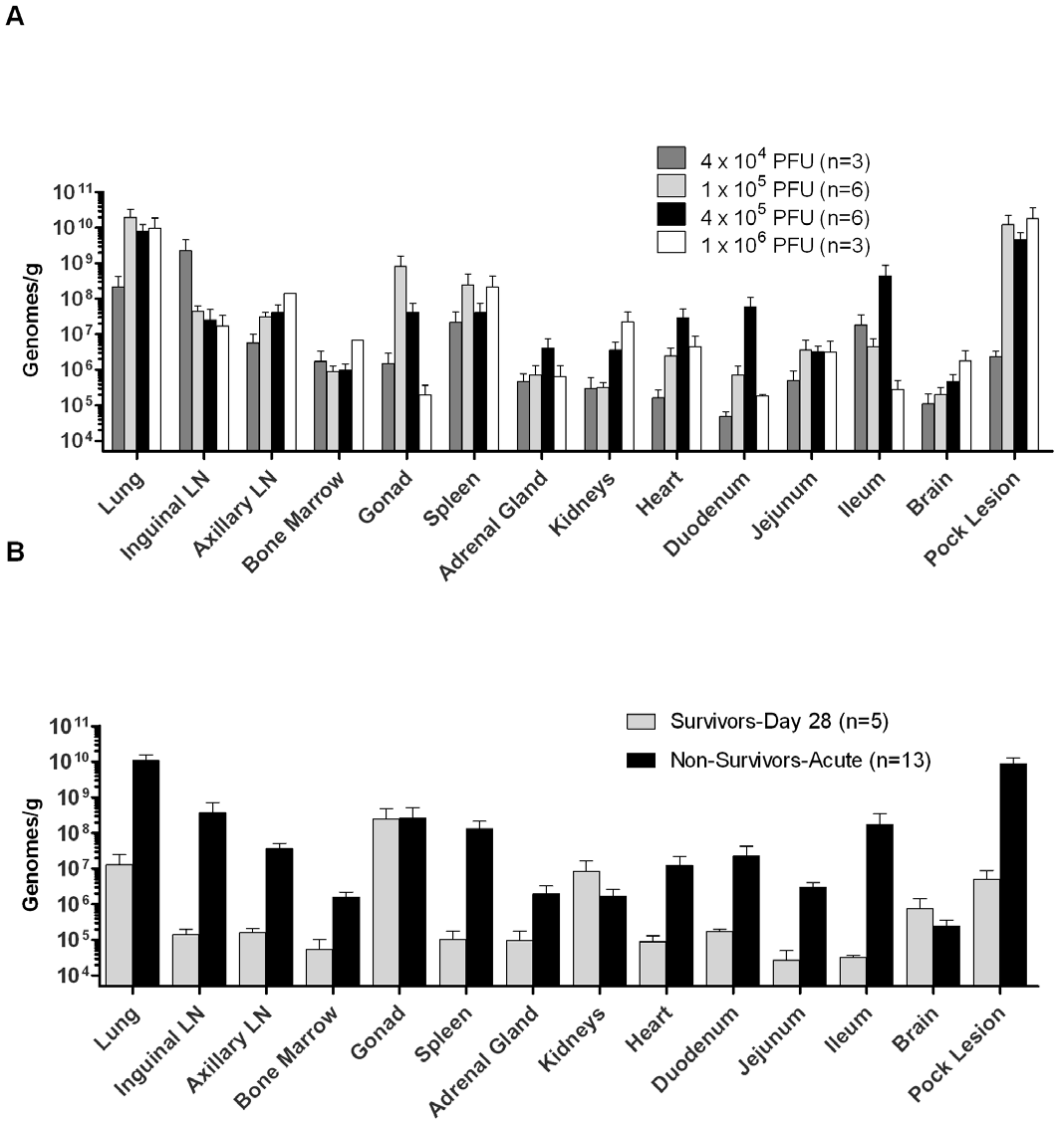
Viral load in tissues. **A**) Tissue viral load of macaques exposed to different doses of aerosolized MXPV and euthanized during the acute phase of disease (survivors are not included). **B**) Tissue viral load in survivors and non-survivors. The X-axis is at the limit of detection = 5000 genomes/g; n: number of animals.

DNA was isolated for real-time PCR from selected tissues of macaques euthanized during acute disease and in those that were euthanized at the end of the study. As expected, lung tissue and pock lesions had the highest viral load for most exposure groups, reaching 10^10^ genomes/g (Fig. 5A). Spleen, gonads, axillary lymph nodes, and inguinal lymph nodes also showed high viral loads in all groups. Viral loads were much higher in the tissues from macaques euthanized during acute illness, in contrast to those that were convalescing (Fig. 5B). Virus persisted in gonads and kidneys but was reduced in other organs of survivors 28 days post-exposure.

### Pathology

Gross and histopathologic features of animals euthanized during acute disease (days 8–11 post-exposure) were similar to those previously reported for aerosolized MPXV [25]. Key histopathologic findings are presented in Table 2. The cause of death was attributed to primary fibrinonecrotic bronchopneumonia. Necrotizing lesions were also present in the skin, gastrointestinal tract (esophagus, stomach, duodenum, colon), lymphoid organs (tonsil, spleen, thymus, gut-associated lymphoid tissue, lymph nodes), mucosal surfaces (oral cavity, trachea, larynx), and gonads [25]. Additional necrotizing lesions were observed rarely in the prostate gland, uterus, skeletal muscle, urinary bladder, bone marrow, and conjunctiva. MPXV-associated lesions were not observed in the liver. There were mild variations in severity of lesions among the different groups, although these differences were not dose-dependent.

**Table 2:** Key histopathologic lesions in cynomolgus macaques exposed to aerosolized MPXV.

Tissue	4×10^4^ PFU		1×10^5^ PFU		4×10^5^ PFU		1×10^6^ PFU	
Histopathologic Findings	n	%	n	%	n	%	n	%
**lung**bronchopneumonia,								
fibrinonecrotic	2/3	66	4/6	66	5/6	83	2/3	66
pleuritis, necrotizing	3/3	100	4/6	66	3/6	50	2/3	66
discrete foci of necrosis								
/inflammation *	1/3	33	0	0	1/6	16	0	0
perivascular/peribronchial								
inflammation*	0	0	2/6	33	1/6	16	1/3	33
**spleen**								
splenitis, necrotizing	1/3	33	2/6	33	4/6	66	2/3	66
lymphoid depletion	2/3	66	3/6	50	3/6	50	2/3	66
lymphoid hyperplasia*	0	0	0	0	0	0	1/3	33
**mandibular lymph node**								
lymphadenitis, necrotizing	1/3	33	2/6	33	5/6	83	2/3	66
lymphoid depletion	0	0	2/6	33	3/6	50	1/3	33
lymphoid hyperplasia*	1/3	33	2/6	33	2/6	33	1/3	33
**mesenteric lymph node**								
lymphadenitis, necrotizing	0	0	0	0	0	0	1/3	33
lymphoid depletion	0	0	0	0	1/6	16	2/3	66
lymphoid hyperplasia*	0	0	2/6	33	1/6	16	0	0
**axillary lymph node**								
lymphadenitis, necrotizing	0	0	3/6	50	3/6	50	2/3	66
lymphoid depletion	0	0	2/6	33	0	0	2/3	66
lymphoid hyperplasia*	1/3	33	2/6	33	2/6	33	1/3	33
**inguinal lymph node**								
lymphadenitis, necrotizing	0	0	1/6	16	2/6	33	2/3	66
lymphoid depletion	0	0	0	0	0	0	2/3	66
lymphoid hyperplasia*	1/3	33	2/6	33	2/6	33	1/3	33
**tracheobronchial lymph node**								
lymphadenitis, necrotizing	2/3	66	2/6	33	4/6	66	1/2	50
lymphoid depletion	2/3	66	3/6	50	3/6	50	0	0
lymphoid hyperplasia*	0	0	1/6	16	1/6	16	1/2	50
discrete foci of necrosis								
/inflammation *	0	0	0	0	0	0	1/2	50

* = lesion seen in survivors**.**

Survivors had a variety of chronic lesions, including discrete, nodular to coalescing areas of necrosis and inflammation in the lung and mediastinal lymph nodes, chronic inflammation centered on bronchi and vessels, type II pneumocyte hyperplasia, pleural and interstitial fibrosis, and fibrous pleural adhesions. Other lesions included lymphoid hyperplasia and plasmacytosis, and chronic periadnexal and perivascular dermatitis.

**Figure 6:**
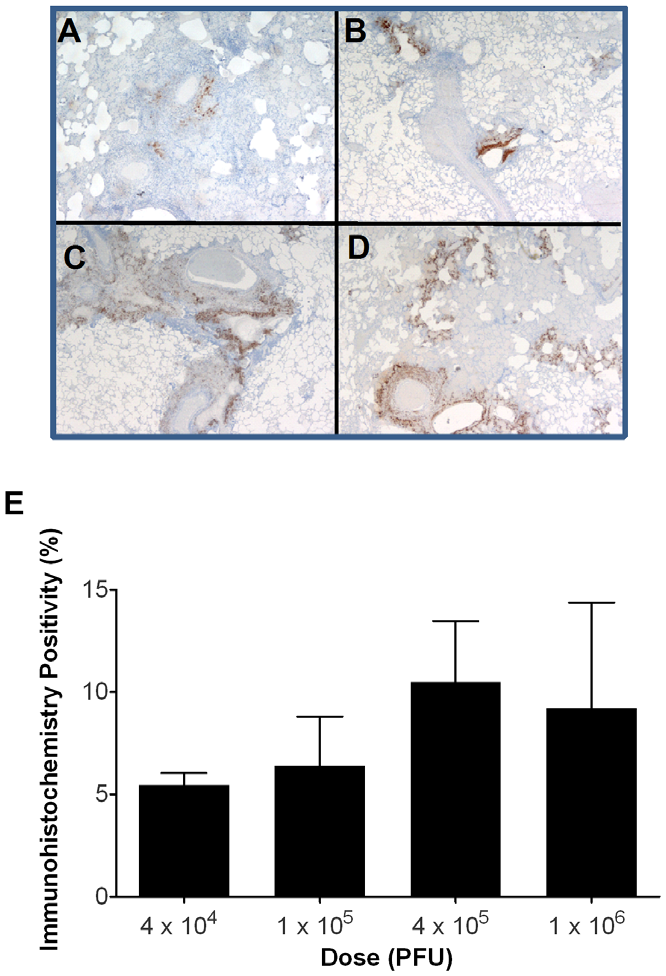
Pathology and presence of MPXV antigen in lung tissue. Figures **A–D** are histological sections of lung tissues from cynomolgus macaques infected via aerosolized MPXV. Positive immunoreactivity for orthopoxvirus antigen, shown as brown staining, is associated with necrotizing lesions primarily concentrated around bronchi and bronchioles. [Immunoperoxidase method using rabbit polyclonal antibody to vaccinia virus; original magnification ×40 (Figure 6A) or ×20 (Figure 6 B, C, D)]. **A**) 4×10^4^ PFU (day 10 post-exposure). **B**) 1×10^5^ PFU (day 8 post-exposure). **C**) 4×10^5^ PFU (day 11 post-exposure). **D**) 1×10^6^ PFU (day 9 post-exposure). **E**) Percent immunoreactivity in the lungs of non-survivors by dosage group, measured by digital microscopy image analysis.

Positive orthopoxvirus immunoreactivity was associated with necrotizing lesions in animals that died during acute disease. In the lungs, this was predominantly concentrated around bronchi and bronchioles (Fig. 6A-D). No poxvirus immunoreactivity was observed in normal uninfected NHP splenic tissue which served as a negative control (data not shown). The percentage of orthopoxviral antigen in lung sections of non-survivors increased with increasing dosage of virus, but the differences were not significant (Fig. 6E). In animals that survived, non cell-associated poxvirus antigen was detected in the center of discrete areas of necrosis in the lung (3/5 animals) and mediastinal lymph node (1/5 animals) - these findings were not dose-dependent.

## Discussion

Different routes of poxvirus exposure can lead to variations in disease course in both humans and monkeys [31], [32]. Many studies used i.v. delivery of MPXV as a model for smallpox and to test vaccines and countermeasures [33]. However, aerosol delivery of MPXV most closely mimics the route of natural transmission of smallpox among humans, which is by the respiratory route [22]. The pathogenesis of aerosol MPXV infection is comparable to smallpox because the infection is initiated in the respiratory mucosa followed by spread to local lymph nodes before primary viremia ensues. A major pitfall of the i.v. MPXV infection model is that the initial infection of respiratory tissue, incubation, and prodromal phases are bypassed with the direct initiation of viremia. The same phenomenon has occurred in human MPXV infections initiated by scratch or bite versus those presumed to have occurred by respiratory exposure [31]. This is an important caveat when the utility of these models is meant to test possible vaccines and treatments in which the efficacy may depend on protecting the respiratory mucosa and targeting subsequent early stages of the infection, which are not represented in the i.v. challenge model.

A marmoset *(Callithrix jacchus)* model of intranasal calpox, a strain of cowpox virus, was recently evaluated [14]. Advantages of this model include the lower lethal dose required and the relative ease of husbandry of marmosets compared to larger species of NHPs. However, there are several disadvantages to the intranasal marmoset calpox model. First, the intranasal route is less physiologically relevant to a naturally occurring poxvirus infection, despite the relative technical ease at which intranasal challenge can be accomplished compared to aerosol challenge. Additionally, the clinical disease course was less similar to that of MPXV or VARV infection of humans, due to the appearance of very few pox lesions and the appearance of observable clinical disease of short duration just prior to death. Finally, perhaps because of the small size of the marmosets, blood was not drawn with great frequency and serum chemistries and CBCs were not performed, thus leaving many features of the clinical disease course unexplored.

An intratracheal infection model deposits virus directly into airways but without regard to particle size and the physiological deposition that occurs during the process of inhalation. Fibrinonecrotic bronchopneumonia was described in animals that received 10^7^ pfu MPXV by i.t. inoculation, as was also the case in animals infected by the aerosol route in this study as well as the study by Zaucha and colleagues [25], [34]. Intratracheal MPXV infection with a comparable dose of virus to that used in this study (10^6^ PFU) resulted in a strikingly similar curve for viremia to that seen in this study, with a large peak followed by a smaller peak at approximately 22 days post exposure [34]. However, the timing of the first peak was delayed by 5 days in i.t. exposed macaques compared to aerosol infection, and the amount of virus detected by qPCR was approximately 100-fold lower. This suggests that local replication is more pronounced after aerosol delivery compared to the i.t. route. Taken together, i.t. inoculation is therefore not a substitute for the aerosol route of infection.

Human MPXV infection has a mortality ranging from 1.5% to 10% according to epidemiologic data, and is clinically indistinguishable from human smallpox except for a greater frequency of lymphadenopathy [3], [35], [36]. Likewise, a 1961 study reported similar diseases after aerosol infection of cynomolgus macaques with MPXV compared to several VARV strains [37]. While MPXV is thought to have a lower potential for human-to-human transmission than VARV, it is currently the most troublesome orthopoxvirus for humans, with sporadic outbreaks occurring most commonly in the African Congo [35]. An outbreak of human monkeypox occurred in the Midwestern US in 2003 after contact with prairie dogs infected with a West African strain of MPXV [38]. MPXV-infected humans had a longer incubation and shorter fever duration than the macaques in this study, which may be due in part to a presumed lower dose of a natural environmental exposure or differences in virulence between the West African strain causing the US outbreak and the Central African strain used for this study [17], [39].

Hematology data indicated that WBC levels were significantly increased from the baseline levels by day 4 post-exposure (*P* = 0.0013), with the WBC levels in some dosage groups increasing above the normal range by day 10. The increased neutrophils and decreased lymphocytes - although not true neutrophilia and lymphopenia, since the values were within reference ranges - would be consistent with an inflammatory leukogram. Similarly, leukocytosis was observed in 45% of human monkeypox cases [38]. Thrombocytopenia was seen in 35% of human patients and significant decreases in platelet levels also occurred on day 6 post-exposure in macaques. Histologic evaluation of bone marrow did not reveal significant pathology; thus, the mechanism of decreased platelets was unlikely due to decreased production but more likely due to increased consumption or removal due to viral infection.

In contrast to the human cases that had low BUN levels (61%), MPXV exposed macaques had normal BUN indicating that decreased production of urea or reduced availability of ammonia for urea synthesis tends to occur in humans but may not occur in MPXV infected macaques [38]. The animals in this study had no pathologic abnormalities in the kidneys despite the persistence of virus in the kidneys of animals surviving infection. Macaques in the 1×10^5^ PFU, 4×10^5^ PFU, and 1×10^6^ PFU groups had significantly elevated AST at day 10 post-exposure compared to day -1 (*P* = 0.002). Likewise, high transaminase levels were detected in 50% of MPXV infected humans. Although AST levels in MPXV infected macaques were within the normal range, the levels were elevated compared to baseline levels.

LDH is a protein that aids in the removal of lactate from tissues. Lactate is the end product when anaerobic glycolysis occurs in low oxygen conditions. Increased LDH in MPX infection indicates increased lactate and therefore decreased oxygen in the body. Since aerosolized MPX infection affects the lungs, this is a possible cause of elevated LDH levels. Additionally, because LDH lacks tissue specificity and the major sources of high serum LDH activity include muscle, liver, and erythrocytes, we cannot rule out these sources as contributing factors. As observed in 50% of MPXV infected humans diagnosed with hypoalbuminemia, significant decreases in albumin levels were also seen in this study in each of the dosage groups by day 6 post-exposure (*P* = 0.0056). Hypoproteinemia and hypoalbuminemia likely resulted from anorexia which may have been the consequence of lesions within the mouth and esophagus. Thus, there are a number of similarities between monkeypox disease in humans and aerosol exposed macaques.

The pathologic findings in this study are similar to those reported by Zaucha et al. [25]. In animals with acute disease, fibrinonecrotic bronchopneumonia was the most distinctive lesion observed. In animals surviving the infection, nodular to coalescing foci of necrosis and inflammation appeared to be centered on large airways. Other lesions in animals with acute disease included necrotizing lesions in the skin, mucosal surfaces, lymphoid tissues, and gonads. Cutaneous lesions ranged from very few in some animals to too numerous to count in others. Additional lesions seen in this study, albeit infrequently, were necrotizing lesions in the prostate gland, uterus, skeletal muscle, and urinary bladder. Hepatic lesions were not observed in our study, whereas Zaucha et al. described disseminated hepatitis in 13% of MPXV infected animals.

Generally, a dose of 10^6^ pfu MPXV given by aerosol to cynomolgus macaques is uniformly lethal and would be an appropriate dose for testing of orthopoxvirus countermeasures (data not shown). However, in this report, one of three animals survived challenge at this dose, possibly due to individual differences in the host immune response. Future studies should be done to determine which primate host responses are amenable to survival following aerosol exposure of MPXV.

Interestingly, the average weight of the survivors was 20% heavier than that of the non-survivors. Macaques were between 4 and 6 years old, therefore age differences were not likely a factor in survival. Of the five animals that survived, four were male, indicating that males, which tend to be heavier, may be more resistant to MPXV. Increased survival in male macaques could be due either to gender or heavier weight. Among humans, correlations of MPXV disease severity with weight, gender, or age have not been made, although pediatric patients were more likely than adults to be admitted into an intensive care unit in the 2003 US outbreak [38].

Unlike other animal models of orthopoxvirus infection, the model presented here utilizes the respiratory route which is the natural route of transmission for human VARV infections and a secondary route for human MPXV infections. We have demonstrated that aerosol infection of cynomolgus macaques with MPXV has a number of parallels to human monkeypox and smallpox diseases. Therefore, an aerosol infection model in NHPs is important for understanding orthopoxvirus pathogenesis as well as for future evaluation of novel vaccine and therapeutic candidates.
